# Cruciferous vegetables lower blood pressure in adults with mildly elevated blood pressure in a randomized, controlled, crossover trial: the VEgetableS for vaScular hEaLth (VESSEL) study

**DOI:** 10.1186/s12916-024-03577-8

**Published:** 2024-09-02

**Authors:** Emma L. Connolly, Alex H. Liu, Simone Radavelli-Bagatini, Armaghan Shafaei, Mary C. Boyce, Lisa G. Wood, Lyn McCahon, Henrietta Koch, Marc Sim, Caroline R. Hill, Benjamin H. Parmenter, Nicola P. Bondonno, Amanda Devine, Kevin D. Croft, Richard Mithen, Seng Khee Gan, Carl J. Schultz, Richard J. Woodman, Catherine P. Bondonno, Joshua R. Lewis, Jonathan M. Hodgson, Lauren C. Blekkenhorst

**Affiliations:** 1 Nutrition and Health Innovation Research Institute, School of Medical and Health Sciences, Royal Perth Hospital Research Foundation, Edith Cowan University, Perth, WA Australia; 2 Centre for Integrative Metabolomics and Computational Biology, School of Science, Edith Cowan University, Joondalup, WA Australia; 3 School of Science, Edith Cowan University, Joondalup, WA Australia; 4 School of Biomedical Science and Pharmacy, New Lambton Heights, University of Newcastle, NSW, Australia; 5 School of Biomedical Sciences, Royal Perth Hospital Unit, University of Western Australia, Perth, WA Australia; 6 Medical School, University of Western Australia, Perth, WA Australia; 7 The Danish Cancer Institute, Copenhagen, Denmark; 8 Liggins Institute, University of Auckland, Auckland, New Zealand; 9 Department of Endocrinology and Diabetes, Royal Perth Hospital, Perth, WA Australia; 10 Department of Cardiology, Royal Perth Hospital, Perth, WA Australia; 11 Flinders Health and Medical Research Institute, Flinders University, Adelaide, SA Australia; 12 Centre for Kidney Research, Children’s Hospital at Westmead, School of Public Health, Sydney Medical School, The University of Sydney, Sydney, NSW Australia

**Keywords:** Cruciferous, Brassica, Vegetables, Hypertension, Blood pressure, Older adults, Glucosinolates, Oxidative stress, Inflammation, Cardiovascular disease, Randomized controlled trial

## Abstract

**Background:**

Higher cruciferous vegetable intake is associated with lower cardiovascular disease risk in observational studies. The pathways involved remain uncertain. We aimed to determine whether cruciferous vegetable intake (active) lowers 24-h brachial systolic blood pressure (SBP; primary outcome) compared to root and squash vegetables (control) in Australian adults with mildly elevated BP (SBP 120–160 mmHg inclusive).

**Methods:**

In this randomized, controlled, crossover trial, participants completed two 2-week dietary interventions separated by a 2-week washout. Cruciferous vegetables were compared to root and squash vegetables (~ 300 g/day) consumed with lunch and dinner meals. Participants were blinded to which interventions were the active and control. Adherence was assessed using food diaries and biomarkers (S-methyl cysteine sulfoxide (SMCSO, active) and carotenoids (control)). Twenty-four-hour brachial ambulatory SBP and secondary outcomes were assessed pre- and post each intervention. Differences were tested using linear mixed effects regression.

**Results:**

Eighteen participants were recruited (median (IQR) age: 68 (66–70); female: *n* = 16/18; mean ± SD clinic SBP: 135.9 ± 10.0 mmHg). For both interventions, 72% participants had 100% adherence (IQR: 96.4–100%). SMCSO and carotenoids were significantly different between interventions (mean difference active vs. control SMCSO: 22.93 mg/mL, 95%CI 15.62, 30.23, *P* < 0.0001; carotenoids: − 0.974 mg/mL, 95%CI − 1.525, − 0.423, *P* = 0.001). Twenty-four-hour brachial SBP was significantly reduced following the active vs. control (mean difference − 2.5 mmHg, 95%CI − 4.2, − 0.9, *P* = 0.002; active pre: 126.8 ± 12.6 mmHg, post: 124.4 ± 11.8 mmHg; control pre: 125.5 ± 12.1 mmHg, post: 124.8 ± 13.1 mmHg, *n* = 17), driven by daytime SBP (mean difference − 3.6 mmHg, 95%CI − 5.4, − 1.7, *P* < 0.001). Serum triglycerides were significantly lower following the active vs. control (mean difference − 0.2 mmol/L, 95%CI − 0.4, − 0.0, *P* = 0.047).

**Conclusions:**

Increased intake of cruciferous vegetables resulted in reduced SBP compared to root and squash vegetables. Future research is needed to determine whether targeted recommendations for increasing cruciferous vegetable intake benefits population health.

**Trial registration:**

Clinical trial registry ACTRN12619001294145. https://www.anzctr.org.au

**Supplementary Information:**

The online version contains supplementary material available at 10.1186/s12916-024-03577-8.

## Background

Increasing vegetable intake is widely recommended to reduce cardiovascular disease (CVD) risk [1–3]. Historically researched for their anti-cancer properties, one group of vegetables that have been proposed to have superior benefits on CVD are cruciferous vegetables (e.g., arugula, bok choy, broccoli, Brussels sprouts, cabbage, cauliflower, collard greens, horseradish, kale, radish, turnips, and watercress) [4–6]. Whilst these vegetables are commonly consumed globally, cruciferous vegetables typically make up a small proportion of total vegetable intake (5–24%) [7]. Cruciferous vegetable intake was found to be inversely associated with CVD risk in a dose–response meta-analysis of prospective cohort studies [8]. Similar results were observed when objective markers of cruciferous vegetable intake (i.e., urinary thiocyanate) were considered [9]. More research is required to establish any causal pathways through which cruciferous vegetables benefit cardiovascular health.

Hypertension is the leading risk factor for CVD with its prevalence increasing with age [10]. Glucosinolates are found almost exclusively in cruciferous vegetables and have been shown to lower blood pressure in animals, but evidence in humans is limited [4]. These compounds have been proposed to exhibit cardiovascular health benefits, such as reduced glycemic-related complications, improved endothelial function, and reduced formation and progression of atherosclerotic plaques [4]. Additionally, cruciferous vegetables also contain several other components that likely influence blood pressure, such as nitrate and vitamin K [11, 12].

Few intervention studies have been conducted in humans to investigate the effects of cruciferous vegetables on risk factors for CVD, such as elevated blood pressure. As previously described in the published protocol [13], the primary objective of the VEgetableS for vaScular hEaLth (VESSEL) study was to determine if daily consumption of cruciferous vegetables results in lower 24-h brachial systolic blood pressure (SBP) in middle-aged and older adults with mildly elevated blood pressure compared to root and squash vegetables. Our a priori hypothesis was that daily consumption of cruciferous vegetables, in comparison to root and squash vegetables, would result in a greater reduction in ambulatory blood pressure. Secondary objectives were to determine if cruciferous vegetables were superior in improving other brachial and arterial ambulatory blood pressures, arterial stiffness, and circulating biomarkers of oxidative stress and inflammation and other CVD risk factors.

## Methods

### Ethics

The Edith Cowan University Human Research Ethics Committee granted ethics approval for the VESSEL Study (2019–00356-BLEKKENHORST) and the trial was registered at www.anzctr.org.au (ACTRN12619001294145). Written informed consent was obtained from all participants. Procedures were followed in accordance with institutional guidelines.

### Study design

**Fig. 1:**
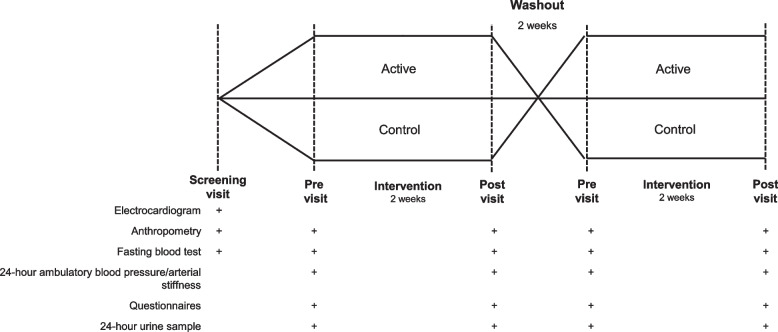
Overview of study design

The VESSEL study was a randomized, controlled, crossover trial with two 2-week dietary intervention periods, as previously described in the protocol [13]. Intervention periods were separated by a 2-week washout (Fig. 1). The study was conducted at the Royal Perth Hospital Research Foundation, Perth, Australia.

### Participants

Six newspaper advertisements were placed at varying intervals between August 2019 and March 2021 to recruit men and women aged 50 to 75 years with mild to moderately elevated blood pressure (SBP 120–160 mmHg, inclusive, and diastolic (DBP) < 100 mmHg) from the general population of Perth, Australia. Detailed inclusion and exclusion criteria are shown in Additional File 1: Table S1 [2, 14].

Screening blood pressure was assessed using a CARESCAPE Dinamap v100 Vital Signs Monitor (GE Healthcare, Buckinghamshire, UK). After resting in a supine position for 5 min, five blood pressure and heart rate measurements were taken at 1-min intervals. The first measurement was excluded, and the next four readings were averaged to calculate mean resting blood pressure.

### Randomization

Using computer-generated random numbers, eligible participants were randomly assigned to one of two intervention sequence orders (1:1 allocation). The intervention sequence orders were placed in opaque sealed envelopes by a study investigator and opened in consecutive order as participants were enrolled in the study.

### Dietary intervention

Participants completed two 2-week dietary interventions in random order, as follows:

1. Active: four serves (~ 300 g/day) of cruciferous vegetables (broccoli, kale, cauliflower, and cabbage) consumed as two soups: one at lunch and one at dinner (~ 600 mL soup/day, ~ 600 kJ/day).
2. Control: four serves (~ 300 g/day) of root and squash vegetables (potato, sweet potato, carrot, and pumpkin) consumed as two soups: one at lunch and one at dinner (~ 600 mL soup/day, ~ 600 kJ/day).

All soups were prepared at Edith Cowan University, Joondalup Campus, Perth, Australia, using standardized recipes, as detailed in the protocol [13]. Vegetables were chopped and boiled prior to blending into a soup and were immediately frozen at − 18 °C for storage. Soup was generally consumed within the week, but could be stored for up to 6 weeks at − 18 °C. The active soup contained 40% broccoli, 25% cauliflower, 25% cabbage, and 10% kale, and the control soup contained 40% potato, 30% pumpkin, 20% carrot, and 10% sweet potato. Root and squash vegetables were chosen as the control intervention as these vegetables are commonly consumed in Australia [15]. The macronutrient content of the soups was closely matched, as previously reported in the protocol [13]. Participants were instructed not to add salt to their soups and were blinded to which interventions were the active and control.

Standard lunch and dinner meals were provided throughout both interventions to minimize background diet variation amongst participants. These meals provided approximately 1–4 serves (75–450 g) of vegetables per day, excluding the additional vegetable serves provided in the soups. Cruciferous vegetables were avoided when selecting these meals by checking the listed ingredients. Participants were able to select meals based on personal preference and meal orders were duplicated for each intervention to limit variation in the diet between intervention periods. All meals for each participant were ordered and stored at − 18 °C at the beginning of the study period to mitigate potential disruptions to stock availability due to the COVID-19 pandemic.

Participants were instructed to consume their usual breakfast foods and snacks but were asked to avoid consuming any snacks in the 2-h window after soup was consumed. Participants were required to complete food diaries, including the timing of all meals and snacks. Participant food diaries were checked by a dietitian (ELC) and Foodworks software using the AusBrands 2019 and AusFoods 2019 databases was used for dietary assessment (FoodWorks 10 Professional, v10.0. Brisbane: Xyris Pty Ltd, 2019). Intervention adherence was assessed using self-reported (i.e., food diaries) and objective biomarkers of intake (i.e., serum carotenoids and urinary and plasma S-methyl cysteine sulfoxide (SMCSO)). Percent self-reported adherence was calculated by dividing the number of soups consumed by the number of total soups that should have been consumed (28 soups per intervention) and multiplying that number by 100. Urinary and plasma SMCSO were used as objective markers of adherence to the active intervention as SMCSO is found in higher concentrations in cruciferous vegetables, but not root and squash vegetables [16]. Conversely, root and squash vegetables contain higher concentrations of carotenoids than cruciferous vegetables; therefore, serum carotenoids were measured as an objective marker of control intervention adherence [17]. Please see “Biochemical analyses” for methodology used for objective measures of adherence.

### Baseline dietary assessment

To assess baseline habitual dietary intake, participants completed the Dietary Questionnaire for Epidemiological Studies (DQES v3.2), a validated food frequency questionnaire, under the supervision of a trained dietitian or nutritionist (ELC, CRH, BHP) [18]. A dietitian (ELC) looked at outliers for implausible energy intakes with respect to factors such as BMI, sex, and age and reviewed for unrealistic energy intakes to support body function and lifestyle. All vegetables (including legumes and potatoes cooked without fat) were included in the analysis of baseline total vegetable intake (g/day). Intake of Asian greens (e.g., bok choy), coleslaw, Brussels sprouts, cauliflower, and broccoli (i.e., available cruciferous vegetables in the DQES v3.2) were combined to create the cruciferous vegetable (g/day) variable for baseline analysis of cruciferous vegetable intake.

### Outcome measurements

#### Ambulatory blood pressure and arterial stiffness

Trained study investigators (AHL, ELC) fitted participants with the Oscar 2 Ambulatory Blood Pressure Monitor (ABPM) system (SunTech Medical Inc., Morrisville, NC, USA) to assess 24-h ambulatory blood pressure at pre- and post-intervention visits (i.e., beginning and end of each 2-week period), as previously described [13]. The ABPM system measured brachial and aortic blood pressure every 20 min during daytime hours (6 am until 10 pm) and every 30 min during nighttime hours (10 pm until 6 am). Participants used the same ABPM for all visits. Participants were instructed to avoid vigorous activity whilst wearing the monitor and to continue with regular daily activities. Participants were excluded if they were missing more than 20% of measurements or if there were more than four hours with no blood pressure measurements. Ambulatory arterial stiffness was assessed using the aortic augmentation index (AIx, %) [19]. The ambulatory AIx data was obtained using the SphygmoCor component of the Oscar 2 ABPM at pre- and post-intervention visits.

#### Anthropometry

Body composition (weight, height, body mass index (BMI), waist and hip circumference, body fat mass) was assessed according to standard protocols at each pre- and post-intervention visit [13].

#### Lifestyle

Online self-administered questionnaires were used to assess lifestyle factors known to influence blood pressure. These factors included physical activity (assessed using the Community Healthy Activities Model Program for Seniors (CHAMPS) [20]) and stress (assessed using the Perceived Stress Scale (PSS) [14]). CHAMPS and PSS questionnaires were completed pre- and post-intervention to assess any changes in physical activity and stress levels, as these lifestyle factors have been shown to influence blood pressure [21].

#### Biochemical analyses

Blood and urine samples were collected at pre- and post-intervention visits. Fasting blood samples were collected by venipuncture into serum-separating tubes (SST) and ethylenediaminetetraacetic acid (EDTA)-coated vacutainers. SST vacutainers were immediately covered in aluminum foil to protect the tubes from light, due to the light sensitivity of carotenoids [17], and sat upright for 30 min prior to centrifugation. Whole blood was centrifuged at 4 °C at 3500 rpm for 10 min. All aliquots were stored at − 80 °C until analysis. Twenty-four-hour urine samples were collected in sterilized containers the day before pre- and post-intervention visits. After participants returned their sample to the clinic, samples were weighed and urine aliquots were stored at − 80 °C until analysis.

SMCSO was measured in urine and plasma samples. Sulforaphane, the isothiocyanate of the glucosinolate, glucoraphanin, was also measured in plasma samples. For the urinary SMCSO analysis, urine samples were thawed on ice and 50 µL of samples were diluted with liquid chromatography mass spectrometry (LC–MS) grade water (1:2 dilution). The diluted samples (50 µL) were transferred to a 1.5 mL centrifuge tube. The acetonitrile (ACN) solution (150 µL) containing 2 µg/mL internal standards (SMCSO-d3) was added, and the samples were vortexed for 2 min and centrifuged at 14,000 rpm and 4 °C for 10 min. A 100 μL aliquot of supernatant was transferred to a 2 mL high-performance liquid chromatography (HPLC) vial for analysis using liquid chromatography tandem mass spectrometry (LC–MS/MS). Chromatographic separation was performed on an UltiMate 3000 Liquid Chromatograph (Thermo Scientific, CA, USA) coupled to a Thermo Scientific TSQ Quantiva Triple Quadrupole Mass Spectrometer equipped with an electrospray ionization (ESI) source. The optimal separation was achieved on ACQUITY UPLC BEH Amide column (100 mm × 2.1 mm ID; Waters) with 1.7 μm particles and a mobile phase of water and ACN containing 10 mM ammonium formate and 50 nM formic acid at pH 3. The flow rate was 0.5 mL/min and the column temperature was maintained at 35 °C with an injection volume of 4 µL. The detection was performed in positive mode (3500 V) and the spectra were acquired in multiple reaction monitoring (MRM) mode. Argon gas was selected as the collision gas and nitrogen as the nebulizer and heater gas.

For the plasma SMCSO and sulforaphane analysis, plasma samples were thawed on ice and 50 µL of samples were transferred to a 1.5 mL centrifuge tube. The methanol solution (150 μL) containing 0.75 µg/mL internal standards (SMCSO-d3 and SFN-d8) was added, and the samples were vortexed for 2 min and centrifuged at 4000 rpm and 4 °C for 10 min. An 80 μL aliquot of supernatant was transferred to a 2 mL HPLC vial for analysis using the LC–MS/MS. The optimal separation was achieved on XBridge C18 column (100 × 3.0 mm packed with 3.5µm particles; Waters) and using a mobile phase of water and ACN both containing 0.1% formic acid. The flow rate was 0.4 mL/min and the column temperature was maintained at 35 °C with an injection volume of 6 µL. As with the urine analysis, the detection was performed in positive mode (3500 V) and the spectra were acquired in MRM mode. Argon gas was selected as the collision gas and nitrogen as the nebulizer and heater gas.

Serum carotenoids were measured using HPLC, as previously described [22]. Briefly, ethanol, ethyl acetate, and hexane were used to extract carotenoids, with canthaxanthin as an internal standard. Dichloromethane:methanol (1:2 vol) was used to reconstitute the dried extract after evaporation of the solvents. Using a Hypersil ODS column (100 mm × 2.1 mm × 5 μm) (Thermo Scientific, CA, USA), chromatography was performed on an Agilent 1200 HPLC system (Agilent Technologies, CA, USA). A mobile phase of ACN:dichloromethane:methanol 0.05% ammonium acetate (85:10:5 vol:vol) at a flow rate of 0.3 mL/min with the use of a diode array detector (450 nm) was used to analyze carotenoids [22].

Plasma F_2_-isoprostanes, a biomarker of oxidative lipid damage, were measured using electron-capture negative-ion gas chromatography–mass spectrometry as total (free plus esterified) F_2_-isoprostanes, as previously described [23]. Serum high sensitivity interleukin-6 (hsIL-6), a marker of inflammation, was analyzed using a commercially available enzyme-linked immunosorbent assay (ELISA) kit (R&D Systems, Inc., Minneapolis, MN, USA). Urinary concentrations of creatinine, sodium, and potassium (markers of sodium and potassium intakes) and serum concentrations of triglycerides, total cholesterol, high-density lipoprotein (HDL) cholesterol, calculated low-density lipoprotein (LDL) cholesterol, glucose, creatinine, high sensitivity C-reactive protein (hsCRP), sodium, and potassium were analyzed by PathWest Laboratories (Fiona Stanley Hospital, Perth, Australia).

### Statistical analysis

#### Sample size

Based on 24-h ambulatory SBP as the primary outcome, a desired sample size of 25 participants was calculated. This sample size was calculated to provide > 90% power to detect a 2.5 mmHg difference in mean 24-h ambulatory SBP, assuming a standard deviation (SD) of 14 mmHg for SBP, a within-period correlation between SBP measurements of 0.6, a between period correlation of mean SBP of 0.6, and a minimum of 40 blood pressure measurements over each 24-h period [24], as described in the published protocol [13]. The estimated change of 2.5 mmHg was based on plausible values for changes in SBP following a nutritional intervention, such as those described previously from the ingestion of black tea [24], as well as also constituting a clinically meaningful change.

#### Statistical methods

All data were analyzed using IBM SPSS Statistics for Windows, version 29.0 (IBM Corp., Armonk, NY, USA) and STATA, version 15.1 (Statacorp, College Station, TX, USA). Statistical significance was set at a two-sided type 1 error rate of *P* < 0.05. The Shapiro–Wilk normality test was used to assess the normality of distributions of continuous variables. Descriptive statistics of normally distributed variables are expressed as mean ± SD, non-normally distributed continuous variables as median and interquartile range (IQR), and categorical variables as number and proportion (%). Paired *t* tests and Wilcoxon signed rank tests were used to compare pre- and post-intervention measurements within the intervention groups for normally and non-normally distributed variables, respectively.

The primary analyses were conducted according to a modified intention-to-treat protocol, including all participants for which pre-intervention visit data for both interventions were obtained. Secondary per-protocol analyses were conducted on participants who completed the study and had complete data. Intention-to-treat and per-protocol analyses were the same for all outcomes except for hsCRP (extreme outlier removed) and ambulatory aortic blood pressure and arterial stiffness (> 10% missing data). Differences between treatments for ambulatory brachial and aortic blood pressure and arterial stiffness were tested using linear mixed effects regression with fixed effects for treatment, pre vs. post treatment, hour, intervention order, and a treatment X pre-post interaction. In this analysis, “hour” referred to the blood pressure readings aggregated for each hour over the 24-h period. Participant ID was included as a random intercept with a random slope for treatment and pre- vs. post treatment. Akaike information criterion (AIC) and Bayesian information criterion (BIC) were used to determine the best model fit. For all other outcomes, differences between treatments were tested using the same model without a time variable (hour). Due to ABPM error, there was > 10% missing data for aortic blood pressure (11% missing data) and arterial stiffness (13% missing data), and therefore, multiple imputation was utilized as per the published protocol [13]. Ten imputations were generated using a chained regression equation including the following variables in the imputation model: visit, hour, daytime/nighttime hours, treatment, intervention order, age, sex, weight, height, and screening blood pressure. ELC and LCB had full access to all data in the study and took responsibility for its integrity and the data analysis.

## Results

### Recruitment

**Fig. 2:**
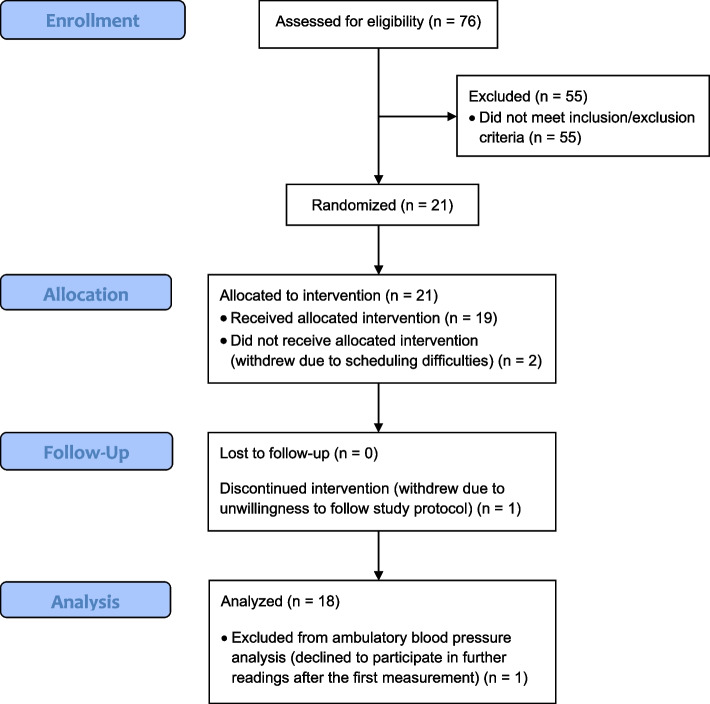
CONSORT diagram

A total of 76 individuals underwent physical screening to be assessed for eligibility. Of these, 21 participants were randomly assigned. Three participants withdrew after randomization: two withdrew due to scheduling difficulties before pre-intervention visit data was collected and one withdrew as they were unwilling to follow study requirements. The CONSORT flow diagram for participant recruitment is shown in Fig. 2. Due to the global COVID-19 pandemic and restrictions put in place in Western Australia, recruitment was paused in March 2020 and later recommenced in October 2020 in a limited capacity.

### Baseline demographic and clinical characteristics

Participants were aged 56 to 72 years and had a BMI range of 21.2 to 35.1 kg/m^2^ (Table 1). Most participants were Caucasian (94%). At screening, participants presented with mean ± SD brachial SBP of 135.9 ± 10.0 mmHg and a DBP of 76.4 ± 7.9 mmHg. Two participants used blood pressure-lowering medication, which remained unaltered throughout the trial. Median (IQR) baseline habitual daily intake of cruciferous vegetable was 26.0 g (18.5–52.9 g) and most people consumed at least 3.5 servings of vegetables per day (Additional File 1: Table S2).

**Table 1:** Demographic and clinical characteristics of study participants at screening/baseline

**Demographic characteristics**	***All participants ***(*n *= 18)
Male/Female, *n* (%)	2 (11%)/16 (89%)
Age, years	68 (66–70)
Marital status, *n* (%)	
-Married	10 (55.6%)
-Single	4 (22.2%)
-Divorced	3 (16.7%)
-De facto	1 (5.6%)
Ethnic background, *n* (%)	
-Caucasian	17 (94%)
-Asian	1 (6%)
BMI, kg/m^2^	28.1 ± 3.9
Ex-smoker, *n* (%)	7 (39%)
Medications	
Blood pressure medication	
-No	16 (88.9%)
-Yes	2 (11.1%)
Clinic blood pressure	
Systolic blood pressure, mmHg	135.9 ± 10.0
Diastolic blood pressure, mmHg	76.4 ± 7.9
Heart rate, beats/min	69.1 ± 8.8
Biochemistry, mmol/L	
Total cholesterol	5.5 ± 0.9
LDL cholesterol	3.2 ± 0.5
HDL cholesterol	1.6 (1.3–1.7)
Triglycerides	1.3 ± 0.5
Glucose	5.5 ± 0.5

Values are mean ± standard deviation, median (IQR), or number (%) as indicated

*Abbreviations*: *HDL *high-density lipoprotein, *IQR *interquartile range, *LDL *low-density lipoprotein

### Dietary intervention

For both soups, 72% of participants had 100% soup adherence (median (IQR) adherence: 100% (96.4–100%) for both interventions). No adverse events were reported. Energy, macronutrient, and food group consumption during both intervention periods is presented in Additional File 1: Table S3. Only protein was significantly different between interventions (*P* = 0.001). Median (IQR) intake of total vegetables per day was 481 g (458–526 g) and 493 g (458–503 g) for the control and active interventions, respectively. Median (IQR) intake of cruciferous vegetables was 0 g/day (0–0 g/day) during the control intervention; four participants reported consuming cruciferous vegetables ranging from 0.3–12.3 g/day during their control intervention. During the active intervention, median (IQR) intake of cruciferous vegetables was 300 g/day (293–300 g/day); three participants reported consuming cruciferous vegetables outside of the intervention soups ranging from 2.1 to 10 g/day during their active intervention.

### Ambulatory blood pressure

**Fig. 3:**
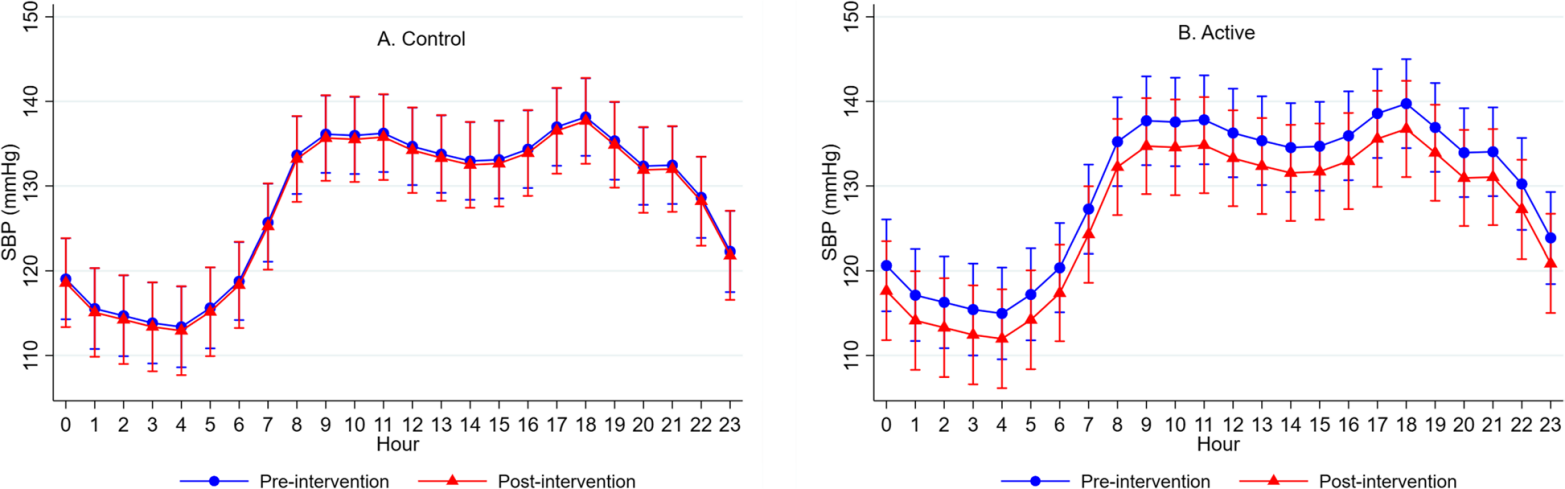
24-h ambulatory systolic blood pressure (SBP) aggregated hourly for the control (**A**) and active (**B**) interventions at the pre- and post-intervention visits

Twenty-four-hour brachial SBP was significantly reduced in the active intervention compared to the control intervention (mean difference active vs. control: − 2.5 mmHg, 95% CI − 4.1, − 0.9, *P* = 0.002) (Table 2). This result was driven by the daytime period (mean difference active vs. control: − 3.6 mmHg, 95% CI − 5.4, − 1.7, *P* < 0.001). No significant difference was seen for nighttime SBP nor 24-h, daytime, and nighttime brachial DBP between interventions (Table 2). Figure 3 shows 24-h brachial SBP at the pre- and post-intervention visit for both interventions. No carryover effects were seen for 24-h brachial SBP (*P* = 0.877) or DBP (*P* = 0.556).

**Table 2:** Ambulatory brachial blood pressure by intervention and differences between interventions

	Intervention (*n* = 17^*^)		
	**Control**	**Active**	**Mean difference (mm Hg) active vs. control (95% CI)**
Overall 24-h SBP, mmHg			
Pre	125.5 ± 12.1	126.8 ± 12.6	
Post	124.8 ± 13.1	124.4 ± 11.8	− 2.5 (− 4.1, − 0.9) *P* = 0.002
Overall 24-h DBP			
Pre	68.8 ± 9.4	69.1 ± 10.8	
Post	68.4 ± 11.2	68.9 ± 9.9	− 0.5 (− 1.7, 0.8) *P* = 0.466
Overall HR, beats/min			
Pre	67.7 ± 10.0	67.5 ± 9.3	
Post	64.4 ± 11.3^†^	66.3 ± 11.4	2.1 (1.1, 3.2) *P* < 0.001
Daytime SBP, mmHg			
Pre	133.1 ± 8.0	135.0 ± 8.6	
Post	133.0 ± 8.7	131.4 ± 6.6	− 3.6 (− 5.4, − 1.7) *P* < 0.001
Daytime DBP, mmHg			
Pre	74.3 ± 6.8	75.4 ± 8.0	
Post	74.0 ± 9.0	74.3 ± 7.4	− 1.0 (− 2.4, 0.4) *P* = 0.149
Daytime HR, beats/min			
Pre	72.1 ± 8.5	72.2 ± 8.0	
Post	69.9 ± 9.9	72.2 ± 9.3	2.2 (0.9, 3.5) *P* = 0.001
Nighttime SBP, mmHg			
Pre	117.9 ± 10.8	118.6 ± 10.6	
Post	116.6 ± 11.6	117.5 ± 11.8	0.7 (− 2.2, 3.5) *P* = 0.653
Nighttime DBP, mmHg			
Pre	63.3 ± 8.6	62.7 ± 9.5	
Post	62.8 ± 10.6	63.6 ± 9.3	1.6 (− 0.5, 3.6) *P* = 0.144
Nighttime HR, mmHg			
Pre	63.3 ± 9.6	62.9 ± 8.2	
Post	58.9 ± 10.0^‡^	60.3 ± 10.4	2.0 (0.6, 3.3) *P* = 0.004

Values are mean ± standard deviation, unless otherwise indicated

*P*-values for pre- vs. post-intervention comparison within the intervention group were obtained using the paired *t* test for normally distributed data. The difference between interventions was tested using linear mixed effects regression with fixed effects for treatment, pre- vs. post treatment, hour, intervention order, and a treatment X pre-post interaction. Participant ID was included as a random intercept with a random slope for treatment and pre vs. post treatment

*CI* confidence interval, *DBP* diastolic blood pressure, *HR* heart rate, *Pre* pre-intervention, *Post* post-intervention, *SBP* systolic blood pressure

^*^One participant was excluded from ambulatory blood pressure analysis due to declining to wear monitor

^†^*P* < 0.05 for pre- vs. post-intervention comparison within the intervention group

^‡^*P* < 0.01 for pre- vs. post-intervention comparison within the intervention group

Between interventions, 24-h and daytime aortic SBP were significantly reduced in the active intervention compared to the control intervention (mean difference active vs. control: − 2.1 mmHg, 95% CI − 3.7, -0.5, *P* = 0.010 and − 3.2 mmHg, 95% CI − 5.0, − 1.4, *P* = 0.001, respectively) (Table 3). Nighttime aortic DBP was significantly increased in the active compared to the control intervention between interventions (mean difference active vs. control: 2.9 mmHg, 95% CI 0.6, 5.2, *P* = 0.014). Ambulatory aortic blood pressure prior to multiple imputations is shown in Additional File 1: Table S4.

**Table 3:** Ambulatory aortic blood pressure and arterial stiffness by intervention and between intervention differences

	Intervention (*n* = 17^*^)		
	**Control**	**Active**	**Mean difference active vs. control (95% CI)**
Overall 24-h AIx (%)			
Pre	37.7 ± 0.5	36.9 ± 0.5	
Post	37.1 ± 0.5	36.8 ± 0.5	0.6 (− 1.2, 2.3) *P* = 0.516
Overall 24-h aortic SBP, mmHg			
Pre	119.9 ± 0.5	120.6 ± 0.5	
Post	120.5 ± 0.5	119.1 ± 0.5^†^	− 2.1 (− 3.7, − 0.5) *P* = 0.010
Overall 24-h aortic DBP, mmHg			
Pre	73.0 ± 0.4	73.7 ± 0.5	
Post	72.8 ± 0.4	73.6 ± 0.4	0.1 (− 1.2, 1.4) *P* = 0.879
Daytime AIx (%)			
Pre	35.4 ± 0.6	34.2 ± 0.6	
Post	35.1 ± 0.6	35.0 ± 0.6	1.2 (− 0.9, 3.4) *P* = 0.271
Daytime aortic SBP, mmHg			
Pre	122.6 ± 0.5	123.5 ± 0.6	
	123.8 ± 0.5	121.3 ± 0.5^‡^	− 3.2 (− 5.0, − 1.4) *P* = 0.001
Daytime aortic DBP, mmHg			
Pre	75.8 ± 0.4	76.9 ± 0.5	
Post	75.9 ± 0.5	76.1 ± 0.5	− 0.7 (− 2.2, 0.7) *P* = 0.325
Nighttime AIx (%)			
Pre	44.5 ± 0.7	44.9 ± 0.8	
Post	43.0 ± 0.8	42.5 ± 0.8^†^	− 1.5 (− 4.5, 1.5) *P* = 0.319
Nighttime aortic SBP, mmHg			
Pre	111.6 ± 1.0	112.1 ± 1.0	
Post	110.2 ± 1.0	112.1 ± 1.0	1.4 (− 1.6, 4.5) *P* = 0.351
Nighttime aortic DBP, mmHg			
Pre	64.8 ± 0.8	64.7 ± 0.9	
Post	63.5 ± 0.8^†^	65.7 ± 0.8	2.9 (0.6, 5.2) *P* = 0.014

Values are mean ± standard error

Within intervention, differences were measured using a mixed effects regression following multiple imputation. The difference between interventions was tested using linear mixed effects regression with fixed effects for treatment, pre vs. post treatment, hour, intervention order, and a treatment X pre-post interaction. Participant ID was included as a random intercept with a random slope for treatment and pre vs. post treatment

*AIx* augmentation index, *CI* confidence interval, *DBP* diastolic blood pressure, *Pre* pre-intervention, *Post* post-intervention, *SBP* systolic blood pressure

^*^One participant was excluded from ambulatory blood pressure analysis due to declining to wear the monitor

^†^*P* < 0.05 for pre- vs. post-intervention comparison within the intervention group

^‡^*P* < 0.01 for pre- vs. post-intervention comparison within the intervention group

Relative to control, there was a significant increase in 24-h and nighttime heart rate for the active intervention (mean difference active vs. control: 2.1 beats/min, 95% CI 1.1, 3.2, *P* < 0.001 and 2.0 beats/min, 95% CI 0.6, 3.3, *P* = 0.004, respectively; Table 2). However, this difference was driven by significant reductions in the control intervention for 24-h (*P* = 0.048) and nighttime heart rate (*P* = 0.004) from pre- to post-intervention visits, which appeared to cause the significant between-group differences.

### Arterial stiffness

The mean difference in AIx was not significantly different between interventions for 24-h, daytime, or nighttime measurements (Table 3). AIx prior to multiple imputations is shown in Supplementary Table 4. No carryover effects were noted (*P* = 0.645).

### Biomarkers of oxidative stress and inflammation

There was no overall difference in the mean F_2_-isprostanes between interventions (Table 4). Plasma F_2_-isoprostanes were significantly lower at the post-intervention visit compared with the pre-intervention visit within the active intervention (*P* = 0.013). However, a similar non-significant drop in F_2_-isprostanes was seen in the control intervention. There was no significant difference in hsCRP and hsIL-6 between interventions (Table 4). One participant was excluded from the per-protocol analysis of hsCRP due to an extreme outlier (hsCRP value: 89.6 mg/L; mean difference active vs. control 0.2 mg/L, 95% CI − 0.9, 1.4, *P* = 0.708). No carryover effects were seen for either F_2_-isprostanes (*P* = 0.901) or hsCRP (*P* = 0.553).

**Table 4:** Biomarkers of oxidative stress and inflammation by intervention and between intervention differences

	**Intervention (*n* = 18)**		
	**Control**	**Active**	**Mean difference active vs. control (95% CI)**
Plasma F_2_-isoprostanes, pmol/L			
Pre	537.66 ± 177.06	518.06 ± 123.83	
Post	475.00 ± 102.40	469.07 ± 88.65^*^	13.67 (− 54.74, 82.09) *P* = 0.695
Serum hsCRP, mg/L			
Pre	2.2 (1.2–3.2)	2.7 (1.6–3.2)	
Post	2.1 (1.2–2.9)	2.0 (1.6–3.0)	5.0 (− 2.7, 12.7) *P* = 0.206
Serum hsIL-6, pg/mL			
Pre	2.9 (2.1–4.4)	2.9 (2.1–3.7)	
Post	3.7 (2.8–4.2)	3.0 (2.7–4.1)	− 0.4 (− 1.3, 0.5) *P* = 0.358

Values are mean ± standard deviation or median (interquartile range)

Differences between treatments were tested using linear mixed effects regression with fixed effects for treatment, pre vs. post treatment, intervention order, and a treatment X pre-post interaction. Participant ID was included as a random intercept with a random slope for treatment and pre vs. post treatment

*CI* confidence interval, *hsCRP* high sensitivity C-reactive protein, *hsIL-6* high sensitivity interleukin 6, *Pre* pre-intervention, *Post* post-intervention

^*^P < 0.05 for pre- vs. post-intervention comparison within the intervention group

### Markers of adherence and metabolism

#### Urinary/plasma SMCSO and plasma sulforaphane

The mean differences in urinary and plasma SMCSO (mean difference active vs. control: 22.93 mg/mL, 95% CI 15.62, 30.23, *P* < 0.0001 and 5.46 mg/mL, 95% CI 4.40, 6.51, *P* < 0.0001, respectively) and plasma sulforaphane concentrations were significantly higher following the active intervention compared to the control intervention (mean difference active vs. control: 0.15 ng/mL, 95% CI 0.06, 0.23, *P* < 0.001) (Table 5). Urinary and plasma SMCSO concentrations, as well as plasma sulforaphane, were significantly higher at the post-intervention visit compared with the pre-intervention visit in the active group (*P* < 0.05 for all) (Table 5).

**Table 5:** Urinary and plasma SMCSO and plasma sulforaphane concentration by intervention and between-intervention differences

	**Intervention (*n* = 18)**		
	**Control**	**Active**	**Mean difference active vs. control (95% CI)**
Urinary SMCSO, mg/mL			
Pre	2.61 (1.93–4.81)	2.58 (1.53–5.04)	
Post	1.83 (1.37–2.89)^*^	22.15 (14.54–25.74)^†^	22.93 (15.62, 30.23) *P* < 0.0001
Plasma SMCSO, mg/mL			
Pre	0.82 (0.40–1.41)	0.52 (0.42, 1.47)	
Post	0.51 (0.36–0.59)^*^	5.30 (4.14–7.64)^†^	5.46 (4.40, 6.51) *P* < 0.0001
Plasma sulforaphane, ng/mL			
Pre	1.05 (1.01–1.15)	1.04 (1.02–1.08)	
Post	1.06 (1.02–1.11)	1.22 (1.12–1.27)^*^	0.15 (0.06, 0.23) *P* = 0.001

Values are median (interquartile range)

*P*-values for pre- vs. post-intervention comparison within the intervention group were obtained using Wilcoxon signed ranks test for non-normally distributed data. The difference between interventions was tested using linear mixed effects regression with fixed effects for treatment, pre- vs post treatment, intervention order, and a treatment X pre-post interaction. Participant ID was included as a random intercept with a random slope for treatment and pre- vs. post treatment

*CI* confidence interval, *Pre* pre-intervention, *Post* post-intervention, *SMCSO* S-methyl cysteine sulfoxide

^*^P < 0.05 for pre- vs. post-intervention comparison within the intervention group

^†^*P* < 0.001 for pre- vs. post-intervention comparison within the intervention group

### Serum carotenoids

Mean differences in total carotenoids, lutein, lycopene, a-carotene, and b-carotene were significantly higher following the control intervention compared to the active intervention (*P* < 0.05 for all) (Table 6). Total and individual carotenoids, excluding b-cryptoxanthin, were significantly higher at the post-intervention visit compared with the pre-intervention visit in the control intervention (*P* < 0.05 for all). In the active intervention, only a-carotene was significantly different at the post-intervention visit compared with the pre-intervention visit.

**Table 6:** Serum carotenoid concentration by intervention and between-intervention differences

	**Intervention (*n* = 18)**		
	**Control**	**Active**	**Mean difference active vs. control (95% CI)**
Total carotenoids, mg/mL			
Pre	1.619 (1.159–1.873)	1.588 (1.307–2.493)	
Post	2.433 (2.249–3.207)^*^	1.885 (1.545–2.693)	− 0.974 (− 1.525, − 0.423) *P* = 0.001
Lutein, mg/mL			
Pre	0.314 (0.244–0.448)	0.308 (0.234–0.567)	
Post	0.446 (0.342–0.576)^*^	0.365 (0.299–0.513)	− 0.090 (− 0.162, − 0.019) *P* = 0.013
B-Cryptoxanthin, mg/mL			
Pre	0.178 (0.139–0.277)	0.183 (0.130–0.280)	
Post	0.198 (0.133–0.318)	0.209 (0.122–0.253)	− 0.028 (− 0.075, 0.019) *P* = 0.237
Lycopene, mg/mL			
Pre	0.653 (0.333–1.020)	0.642 (0.532–0.945)	
Post	1.295 (0.965–1.558)^*^	1.026 (0.668–1.638)^†^	− 0.366 (− 0.725, − 0.008) *P* = 0.045
α-Carotene, mg/mL			
Pre	0.023 (0.017–0.032)	0.032 (0.023–0.041)	
Post	0.048 (0.036–0.061)^*^	0.021 (0.015–0.039)^†^	− 0.037 (− 0.049, − 0.024) *P* < 0.001
b-carotene, mg/mL			
Pre	0.212 (0.159–0.352)	0.363 (0.232–0.476)	
Post	0.611 (0.503–0.738)^*^	0.330 (0.217–0.466)	− 0.452 (− 0.658, − 0.247) *P* < 0.001

Values are mean ± standard deviation or median (interquartile range) as indicated

*P*-values for pre- vs. post-intervention comparison within the intervention group were obtained using Wilcoxon signed ranks test for non-normally distributed data and paired *t* test for normally distributed data. The difference between interventions was tested using linear mixed effects regression with fixed effects for treatment, pre vs. post treatment, intervention order, and a treatment X pre-post interaction. Participant ID was included as a random intercept with a random slope for treatment and pre vs. post treatment

*CI* confidence interval, *Pre* pre-intervention, *Post* post-intervention

^*^P < 0.001 for pre- vs. post-intervention comparison within the intervention group

^†^*P* < 0.05 for pre- vs. post-intervention comparison within the intervention group

### Serum and urinary sodium, potassium, and creatinine

Between interventions, there were no significant mean differences for serum sodium, potassium, or creatine. Urinary sodium, potassium, and creatinine were also not significantly different between interventions (Table 7).

**Table 7:** Biochemical analyses by intervention and between-intervention differences

	**Intervention (*n* = 18)**		
	**Control**	**Active**	**Mean difference active vs. control (95% CI)**
Serum triglycerides, mmol/L			
Pre	1.4 ± 0.6	1.5 ± 0.6	
Post	1.4 ± 0.4	1.3 ± 0.4	− 0.2 (− 0.4, − 0.0) *P* = 0.047
Serum total cholesterol, mmol/L			
Pre	6.0 ± 1.2	6.0 ± 1.0	
Post	5.6 ± 1.0^*^	5.4 ± 0.8^†^	− 0.2 (− 0.5, 0.1) *P* = 0.264
Serum LDL, mmol/L			
Pre	3.7 ± 0.9	3.6 ± 0.8	
Post	3.4 ± 0.9^*^	3.3 ± 0.7^*^	− 0.1 (− 0.4, 0.1) *P* = 0.369
Serum HDL cholesterol, mmol/L			
Pre	1.5 (1.2–1.8)	1.5 (1.3–1.8)	
Post	1.3 (1.1–1.6)^†^	1.3 (1.2–1.6)^†^	0.0 (− 0.1, 0.1) *P* = 0.600
Serum glucose, mmol/L			
Pre	5.4 ± 0.5	5.4 ± 0.5	
Post	5.1 ± 0.4^†^	5.2 ± 0.6^*^	0.1 (− 0.1, 0.3) *P* = 0.357
Serum creatinine, mmol/L			
Pre	67.6 ± 10.7	67.3 ± 11.7	
Post	68.8 ± 11.9	67.8 ± 11.3	− 0.7 (− 2.8, 1.4) *P* = 0.509
Serum sodium, mmol/L			
Pre	138.7 ± 2.0	138.5 ± 1.8	
Post	138.7 ± 1.9	138.4 ± 2.1	− 0.1 (− 0.7, 0.5) *P* = 0.768
Serum potassium, mmol/L			
Pre	4.6 ± 0.3	4.5 ± 0.3	
Post	4.4 ± 0.3	4.4 ± 0.2	0.1 (− 0.1, 0.2) *P* = 0.382
Urinary creatinine, mmol/day			
Pre	9.2 ± 1.7	8.8 ± 1.9	0.3 (− 0.6, 1.2)
Post	8.5 ± 1.6	8.4 ± 1.8	*P* = 0.526
Urinary sodium, mmol/day			
Pre	116.7 ± 39.7	113.2 ± 43.2	− 1.9 (− 26.4, 22.8)
Post	105.7 ± 39.8	100.3 ± 41.1	*P* = 0.883
Urinary potassium, mmol/day			
Pre	73.1 ± 13.2	63.4 ± 21.0	− 5.1 (− 18.3, 8.0)
Post	75.8 ± 16.1	60.9 ± 17.7	*P* = 0.446

Values are mean ± standard deviation or median (interquartile range) as indicated

P-values for pre- vs. post-intervention comparison within the intervention group were obtained using Wilcoxon signed ranks test for non-normally distributed data and paired t test for normally distributed data. The difference between interventions was tested using linear mixed effects regression with fixed effects for treatment, pre vs. post treatment, intervention order, and a treatment X pre-post interaction. Participant ID was included as a random intercept with a random slope for treatment and pre vs. post treatment

*CI* confidence interval, *HDL* high-density lipoprotein, *LDL* low-density lipoprotein, *Pre* pre-intervention, *Post* post-intervention

^*^P < 0.05 for pre- vs. post-intervention comparison within the intervention group

^†^*P* < 0.001 for pre- vs. post-intervention comparison within the intervention group

### Serum lipids and glucose

Serum triglycerides were significantly lower in the active intervention compared to the control intervention (mean difference active vs. control: − 0.2 mmol/L, 95% CI − 0.4, − 0.0, *P* = 0.047) (Table 7). No carryover effects were noted (*P* = 0.7897). There were no significant mean differences between interventions for serum total cholesterol, LDL cholesterol, HDL cholesterol, or serum glucose. However, serum total, LDL, and HDL cholesterol and serum glucose were significantly decreased at the post-intervention visit compared with the pre-intervention visit for both interventions (*P* < 0.05 for all).

### Anthropometry, energy expenditure from physical activity, and stress

There were no significant mean differences between interventions for any anthropometric measures, energy expenditure from physical activity, or perceived stress (*P* > 0.05 for all) (Additional File 1: Table S5). However, weight, BMI, and body fat mass were significantly reduced at the post-intervention visit compared with the pre-intervention visit for both interventions.

## Discussion

In this randomized, controlled, crossover trial, we found that consumption of four serves per day of cruciferous vegetables (active intervention) resulted in a statistically significant reduction in SBP compared with four serves per day of root and squash vegetables (control intervention), supporting our hypothesis. This reduction in SBP is clinically relevant; in a meta-analysis of randomized controlled trials involving pharmacological interventions, a reduction in SBP of 5 mmHg was found to reduce the risk of major cardiovascular events by ~ 10% [25]. Therefore, the 2.5 mmHg reduction in SBP resulting from increasing cruciferous vegetable intake could translate to a 5% lower risk of major cardiovascular events.

Weight reduction is an important component of non-pharmacological management of hypertension [26], with a loss of 1 kg weight associated with approximately 1 mmHg reduction in SBP [27]. Both interventions resulted in a statistically significant reduction in weight post-intervention (control: 1.9 kg; active: 1.3 kg). However, there was no significant difference seen between interventions. Therefore, the improvements in SBP seen with the cruciferous vegetable intervention are likely independent of weight reduction. In addition, there was no significant difference in urinary sodium or potassium excretion between interventions, indicating that the reduction in SBP was independent of dietary sodium and potassium intake.

This blood pressure result is in alignment with other research investigating the breakdown products of cruciferous vegetables, which include glucosinolates and isothiocyanates [28]. Research into the cardio-protective properties of these compounds has largely focused on glucoraphanin, a major glucosinolate found in broccoli, and its isothiocyanate, sulforaphane [5]. Previous studies have investigated the effects of glucosinolates and isothiocyanates on blood pressure in animal models (i.e., rats), with results demonstrating blood pressure-lowering effects [29–32]. However, studies involving humans have been inconsistent. In a study including 40 participants with hypertension (baseline mean blood pressure: control group = 158.6/98; active group = 158.5/96 mmHg), daily ingestion of 10 g dried broccoli sprouts for 4 weeks did not improve blood pressure or endothelial function [33]. Conversely, a 4-week study including participants with type two diabetes and positive for *H. Pylori* (baseline mean blood pressure: standard triple therapy group (*n* = 33) 130/80.4; broccoli sprouts powder group (*n* = 28) 125/80.4; combination group (*n* = 25) 136/89.8 mmHg) found that there was a significant reduction in SBP and DBP in participants who received 6 g/day broccoli sprout powder in combination with standard triple therapy for *H. Pylori* (14 mmHg and 9.4 mmHg reduction in SBP and DBP, respectively) [34]. In a dose escalation study including 12 pregnant women with preeclampsia, activated broccoli extract equivalent to 32 mg or 64 mg of sulforaphane resulted in a trend towards an approximately 10% decrease in DBP (*P* = 0.05) but not SBP [35].

Plasma sulforaphane was significantly higher after the active intervention compared with the control, indicating that glucosinolates (i.e., glucoraphanin) were present in the soup and may explain the beneficial effect seen on blood pressure. These compounds have been proposed to have anti-inflammatory and antioxidant properties due to involvement in increasing Nrf2 activity and inhibition of NF-ĸB [36]. Whilst our findings show that cruciferous vegetable intake did not have a significant effect on our marker of oxidative lipid damage, relative to root and squash vegetables, plasma F_2_-isoprostanes were significantly lower after the active intervention. This highlights the potential efficacy for the antioxidant capabilities of cruciferous vegetables [37], although our study does not provide evidence that this explains the observed difference in SBP. Evidence for cruciferous vegetables altering oxidative stress and inflammation has been inconsistent [38–40] and further studies are needed to investigate the anti-inflammatory and antioxidant capacity of cruciferous vegetables when consumed as part of the diet. Although there were no significant differences in oxidative stress and inflammatory biomarkers between interventions in our study, this could be due to similar benefits of other vegetables in the control treatment. The control soup contained carotenoid-rich vegetables, which also have both antioxidant and anti-inflammatory properties [41]. Furthermore, these biomarkers were within normal expected ranges [42–44], which may also explain this result.

Whilst we report SMCSO as a biomarker of adherence to cruciferous vegetable intake in this study, SMCSO has also been recently identified as a key metabolite associated with the antihypertensive benefits of the Dietary Approaches to Stop Hypertension (DASH) diet [45]. SMCSO contributes a greater proportion of sulfur in cruciferous vegetables than glucosinolates, yet whether SMCSO mediates some of the therapeutic benefits of these vegetables in humans remains largely unexplored [46]. Sulfur-rich vegetables (i.e., cruciferous) are a good source contributing to hydrogen sulfide (H_2_S): the third most important gaseous signaling molecule. As such, this may be a potential mechanism through which these vegetables modulate endothelial function [47]. The contribution of H_2_S to the anti-hypertensive benefits of sulfur-rich allium vegetables (e.g., onion, garlic) has been recently explored [48]; however, more research is required. Both SMCSO and glucosinolates may act as H_2_S donors [45, 47], and the subsequent vasodilation may be partly responsible for the reduction in blood pressure observed in the active intervention.

We also found that serum triglycerides were significantly lower after the active intervention compared with the control intervention. Although pre-clinical evidence suggests that cruciferous vegetables and their glucosinolates may play a role in the reduction of blood lipids [49–52], there is limited evidence in humans. Human trials have shown that broccoli sprouts and glucoraphanin-rich broccoli can improve HDL cholesterol [53] and reduce LDL cholesterol [54], respectively. There were no significant differences in biomarkers of total cholesterol, LDL, HDL, and glucose between interventions in our study. However, this could be due to similar benefits of other vegetables in the control treatment, as there were significant within-group changes in these biomarkers following both interventions.

This study had multiple strengths. First, to our knowledge, our study is the only intervention study in humans to show improvements in blood pressure in middle-aged and older adults with mildly elevated blood pressure after increased short-term consumption of cruciferous vegetables compared to commonly consumed root and squash vegetables. Furthermore, this study is also novel in that the study design included a dietary intervention utilizing a combination of cruciferous vegetables as whole foods (not extracts of cruciferous vegetables or their bioactives). Second, the study had a crossover design which allowed all participants to act as their own control, mitigating potential differences between participants. A 2-week washout period was selected between interventions to avoid potential carryover effects, as this was considered enough time for objective markers of intake to be adequately excreted (i.e., urinary SMCSO in the active intervention and serum carotenoids in the control intervention). This washout period has been used in prior studies, demonstrating that these biomarkers return to normal levels within 2 weeks [55, 56]. Third, in addition to self-reported food diaries, objective markers of intake were measured to corroborate adherence to both dietary interventions. Additionally, we carefully controlled the background diet of participants. The provision of lunch and dinner meals throughout the study likely substantially reduced the background variation in vegetable intake and other foods.

This study also has limitations. Although a significant reduction in our primary outcome of 24-h SBP was observed, we did not reach our desired sample size. The reduced sample size resulting from COVID-19-related issues may be relevant for secondary outcomes in this study. As we were unable to recruit the desired sample size of 25 participants (28 to account for participant drop-out) due to the COVID-19 pandemic, this study may be considered a pilot RCT rather than a phase 2 trial. However, despite this, we were still able to demonstrate a statistically significant result for our primary outcome, brachial 24-h ambulatory SBP, thereby eliminating the possibility of making a type-2 error for the primary outcome. We were aiming to have roughly an equal distribution of males and females; however, this was not possible due to limitations resulting from the COVID-19 pandemic (e.g., logistical and financial) and led to mostly female participants. Most participants were also Caucasian. Therefore, these results may not be as generalizable to males and other ethnicities. Second, participants included in this study had a baseline reported intake of vegetables higher than that of the general population (327 g vs.195 g per day) [57], which may reduce the generalizability of these results. However, this may also be seen as a strength by reinforcing that the type of vegetable consumed matters. Nonetheless, investigation of cruciferous vegetable intake in individuals with lower baseline intake of vegetables is warranted to determine if there is a more profound effect with a greater increase from baseline habitual intake. Third, given the nature of the dietary interventions, participants were unable to be completely blinded, as the different soups had clear differences in color and taste. However, participants were not informed which soup was the active or control or what vegetables were included in each soup. Lastly, self-reported protein intake was significantly different between interventions (7 g higher in the active group in comparison to the control, *P* = 0.001), and increasing protein intake has been shown to influence blood pressure. However, this difference is unlikely to have an effect, based on results of a meta-analysis that indicate amounts of approximately 40 g per day are needed to have a similar effect on blood pressure [58]. It is important to note that cruciferous vegetables do not contain only glucosinolates and SMCSO at higher concentrations than root and squash vegetables. Cruciferous vegetables also contain higher nitrate and vitamin K levels. In addition, they also provide smaller but higher levels of other nutrients and phytochemicals such as magnesium, flavonoids, vitamin C, and folate, all of which have potential to contribute to benefits on blood pressure [11, 12]. As such, we are not able to fully elucidate which specific components are responsible for the beneficial effects that we observed.

## Conclusions

Daily consumption of four serves of cruciferous vegetables over a 2-week period resulted in reduced SBP in middle-aged and older adults with mildly elevated blood pressure compared with root and squash vegetables. Increased intake of a variety of different vegetables has many health benefits due to the presence of vitamins, minerals, and many other bioactive compounds. Future research is needed to inform whether targeted recommendations to increase cruciferous vegetable intake within a healthy diet that includes a variety of vegetables can reduce the public health burden of CVD. Furthermore, the study could be implemented in other regions worldwide to obtain multi-ethnic data.

### Supplementary Information

Additional file 1. Table S1 Detailed inclusion and exclusion criteria. Table S2 Dietary intakes of study participants at baseline obtained using a food frequency questionnaire. Table S3 Comparison of energy, macronutrients, and food groups consumed during both interventions for all participants who completed the study (*n* = 18). Table S4 Ambulatory aortic blood pressure and arterial stiffness by intervention and between intervention differences. Table S5 Anthropometric measurements, energy expenditure from physical activity, and perceived stress by intervention.

## Data Availability

The datasets used and analyzed during the current study are available from the corresponding author on reasonable request.
