# Supplementary material for: Cruciferous vegetables lower blood pressure in adults with mildly elevated blood pressure in a randomized, controlled, crossover trial: the VEgetableS for vaScular hEaLth (VESSEL) study
Source: BMC Med. 2024 Sep 2;22:353. doi: 10.1186/s12916-024-03577-8 (PMC11367748; doi:10.1186/s12916-024-03577-8)
Supplement: Supplementary file 1 — Additional file 1. Table S1 Detailed inclusion and exclusion criteria. Table S2 Dietary intakes of study participants at baseline obtained using a food frequency questionnaire. Table S3 Comparison of energy, macronutrients, and food groups consumed during both interventions for all participants who completed the study (n = 18). Table S4 Ambulatory aortic blood pressure and arterial stiffness by intervention and between intervention differences. Table S5 Anthropometric measurements, energy expenditure from physical activity, and perceived stress by intervention. [file 12916_2024_3577_MOESM1_ESM.docx]

**Cruciferous vegetables lower blood pressure in adults with mildly elevated blood pressure: a randomized, controlled, crossover trial**

**Authors:** Emma L. Connolly^1^, Alex H. Liu^1^_,_ Simone Radavelli-Bagatini^1^, Armaghan Shafaei^2^, Mary C. Boyce^3^, Lisa G. Wood^4^, Lyn McCahon^1^, Henrietta Koch^5^, Marc Sim^1,6^, Caroline R. Hill^1^, Benjamin H. Parmenter^1^, Nicola P. Bondonno^1,7^, Amanda Devine^1^, Kevin D. Croft^5^, Richard Mithen^8^, Seng Khee Gan^6,9^, Carl J. Schultz^6,10^, Richard J. Woodman^11^, Catherine P. Bondonno^1,6^, Joshua R. Lewis^1,6,12^, Jonathan M. Hodgson^1,6^, Lauren C. Blekkenhorst^1,6^

**Author affiliations:** ^1^Nutrition and Health Innovation Research Institute, School of Medical and Health Sciences, Edith Cowan University, Joondalup, Western Australia, Australia; ^2^Centre for Integrative Metabolomics and Computational Biology, School of Science, Edith Cowan University, Joondalup, Western Australia, Australia; ^3^School of Science, Edith Cowan University, Joondalup, Western Australia, Australia; ^4^School of Biomedical Science and Pharmacy, University of Newcastle, New Lambton Heights, New South Wales, Australia; ^5^School of Biomedical Sciences, Royal Perth Hospital Unit, University of Western Australia, Perth, Western Australia, Australia; ^6^Medical School, University of Western Australia, Perth, Western Australia, Australia; ^7^The Danish Cancer Institute, Copenhagen, Denmark; ^8^Liggins Institute, University of Auckland, Auckland, New Zealand; ^9^Department of Endocrinology and Diabetes, Royal Perth Hospital, Perth Western Australia; ^10^Department of Cardiology, Royal Perth Hospital, Perth, Western Australia; ^11^Flinders Health and Medical Research Institute, Flinders University, Adelaide, South Australia, Australia; ^12^Centre for Kidney Research, Children's Hospital at Westmead, School of Public Health, Sydney Medical School, The University of Sydney, Sydney, New South Wales, Australia

**Table S1 Detailed inclusion and exclusion criteria**

| **Inclusion criteria** |
| --- |
| - Ambulant community-dwelling men and women - Aged between 50 and 75 years - Mildly elevated blood pressure (systolic blood pressure 120–160 mmHg, inclusive, and diastolic blood pressure <100 mmHg) |
| **Exclusion criteria** |
| - Systolic blood pressure >160 mmHg or <120 mmHg - Diastolic blood pressure ≥100 mmHg - Use of >2 antihypertensive medications or irregular use of nitric oxide donors, organic nitrites and nitrates, and sildenafil and related drugs - Diagnosed diabetes or fasting blood glucose >6.5 mmol/L - Fasting total cholesterol >8 mmol/L - Body mass index <18.5 or ≥40 kg/m2 - Current or recent (<12 months) smoking - Adhesive allergy - Regular aspirin use - Medication use for thrombosis or anticoagulants (Warfarin) - History of cardiovascular or peripheral vascular disease (myocardial infarction, stroke, transient ischemic attack, amputation due to arterial insufficiency, any form of arterial revascularization, history of exertional angina or claudication) - Psychiatric illness or other major illnesses, such as cancer - Alcohol intake >100 g per week - Current or recent (within previous 6 months) significant weight loss or gain (>6% of body weight) or actively trying to lose weight - Pre-menopausal women - Inability to attend clinic/office visits - Use of antibiotics (within previous 2 months) - Use of antibacterial mouthwash and not willing to cease for trial duration - Reported participation in night shift work during the study period - Inability or unwillingness to follow the study protocol - Specific dietary requirements, allergies, or intolerances that will interfere with their ability to follow the dietary requirements |

**Table S2 Dietary intakes of study participants at baseline obtained using a food frequency questionnaire**

| **Dietary intake** | **Participants (n = 17)^1^** |
| --- | --- |
| Energy intake, kJ/day | 7547 ± 1512 |
| Protein, g/day | 83.8 ± 18.2 |
| Total fat, g/day | 85.8 ± 25.9 |
| Saturated fat, g/day | 24.9 (20.1-31.6) |
| Monounsaturated fat, g/day | 35.0 ± 12.8 |
| Polyunsaturated fat, g/day | 15.4 ± 6.1 |
| Carbohydrate, g/day | 146.6 ± 38.3 |
| Sugars, g/day | 80.0 ± 19.9 |
| Starch, g/day | 64.8 ± 22.8 |
| Fiber, g/day | 23.5 ± 6.7 |
| Alcohol, g/day | 1.4 (0.2-9.1) |
| Total vegetables^2^, g/day | 327.3 (266.4-367.6) |
| Cruciferous vegetables, g/day | 26.0 (18.5-52.9) |
| Total fruit^2^, g/day | 194.6 (65.0-284.2) |
| Grain (cereal) foods^2^, g/day | 95.0 ± 47.2 |
| Meat or alternatives^2^, g/day | 225.0 (204.6-269.7) |
| Dairy or alternatives^2^, g/day | 286.0 ± 202.5 |
| Values are mean ± standard deviation or median (interquartile range) as indicated.  ^1^One participant was excluded for implausible energy intake.  ^2^Variables were calculated according to the Australian dietary guidelines (2). | |

**Table S3** **Comparison of energy, macronutrients, and food groups consumed during both interventions for all participants who completed the study (n = 18)**

| **Intake** | **Control** | **Active** |
| --- | --- | --- |
| Energy, kJ/day | 6257 ± 1709 | 6186 ± 1506 |
| Protein, g/day | 56.3 ± 10.6 | 63.3 ± 11.0^1^ |
| Total fat, g/day | 48.3 ± 26.7 | 46.1 ± 20.4 |
| Saturated fat, g/day | 19.4 ± 11.1 | 19.0 ± 8.7 |
| Carbohydrate, g/day | 193.0 ± 40.3 | 185.2 ± 36.5 |
| Alcohol, g/day | 0.0 (0.0-1.8) | - 1. (0.0-6.2) |
| Vegetables^2^, serves/day | 6.4 (6.1-7.0) | 6.6 (6.1-6.7) |
| Fruit^3^, serves/day | 1.0 ± 0.6 | 1.0 ± 0.6 |
| Grain (cereal) foods^3^, serves/day | 6.1 ± 1.1 | 6.1 ± 1.0 |
| Meat or alternatives^3^, serves/day | 1.5 ± 0.6 | 1.6 ± 0.6 |
| Dairy or alternatives^3^, serves/day | 1.2 ± 0.8 | 1.4 ± 0.9 |
| Values are mean ± standard or median (interquartile range) as indicated.  ^1^P = 0.001 (paired t test).  ^2^Vegetable serves were calculated according to the Australian dietary guidelines (2), with one serve = 75 g.  ^3^Variables were calculated according to the Australian dietary guidelines (2) using Foodworks software. | | |

**Table S4 Ambulatory aortic blood pressure and arterial stiffness by intervention and between intervention differences**

|  | **Intervention (n = 14^1^)** | | |
| --- | --- | --- | --- |
|  | **Control** | **Active** | **Mean difference active vs. control (95% CI)** |
| **Overall 24-hour AIx (%)** |  |  |  |
| Pre | 39.9 ± 7.3 | 39.8 ± 7.9 |  |
| Post | 38.2 ± 7.0 | 37.8 ± 5.9 | 0.3 (-1.7, 2.3)  P = 0.767 |
| **Overall 24-hour aortic SBP, mmHg** |  |  |  |
| Pre | 115.7 ± 10.3 | 117.6 ± 10.7 |  |
| Post | 116.1 ± 11.9 | 116.0 ± 9.8 | -1.1 (-2.9, 0.6)  P = 0.211 |
| **Overall 24-hour aortic DBP, mmHg** |  |  |  |
| Pre | 68.8 ± 8.9 | 69.7 ± 11.0 |  |
| Post | 68.0 ± 10.9 | 69.8 ± 9.9 | 0.5 (-0.9, 1.9)  P = 0.506 |
| **Daytime AIx (%)** |  |  |  |
| Pre | 35.3 ± 5.4 | 34.2 ± 5.4 |  |
| Post | 34.6 ± 7.2 | 34.6 ± 5.0 | 1.0 (-1.4, 3.4)  P = 0.417 |
| **Daytime aortic SBP, mmHg** |  |  |  |
| Pre | 121.7 ± 6.6 | 123.3 ± 8.2 |  |
| Post | 121.7 ± 7.3 | 120.7 ± 5.8 | -1.7 (-3.7, 0.3)  P = 0.090 |
| **Daytime aortic DBP, mmHg** |  |  |  |
| Pre | 74.7 ± 5.7 | 75.8 ± 8.2 |  |
| Post | 73.7 ± 7.2 | 75.1 ± 7.2 | 0.2 (-1.4, 1.8)  P = 0.777 |
| **Nighttime AIx (%)** |  |  |  |
| Pre | 44.4 ± 6.2 | 45.3 ± 5.8 |  |
| Post | 41.7 ± 4.8^2^ | 41.0 ± 4.9^3^ | -1.8 (-5.0, 1.3)  P = 0.256 |
| **Nighttime aortic SBP, mmHg** |  |  |  |
| Pre | 109.7 ± 10.0 | 111.9 ± 10.0 |  |
| Post | 110.6 ± 13.3 | 111.4 ± 10.9 | 0.8 (-2.4, 4.0)  P = 0.631 |
| **Nighttime aortic DBP, mmHg** |  |  |  |
| Pre | 62.9 ± 7.5 | 63.6 ± 10.2 |  |
| Post | 62.2 ± 11.1 | 64.4 ± 9.4 | 1.8 (-0.7, 4.3)  P = 0.160 |
| Values are mean ± standard deviation.  The difference between interventions was tested using linear mixed effects regression with fixed effects for treatment, pre versus post treatment, hour, intervention order, and a treatment X pre-post interaction. Participant ID was included as a random intercept with a random slope for treatment and pre versus post treatment.  ^1^Measured in 14 participants as four participants did not have baseline data for both interventions.  ^2^P < 0.05 for pre- vs. post-intervention comparison within the intervention group.  ^3^P < 0.01 for pre- vs. post-intervention comparison within the intervention group.    Abbreviations: AIx, augmentation index; CI, confidence interval; Pre, pre-intervention; Post, post-intervention | | | |

**Table S5 Anthropometric measurements, energy expenditure from physical activity, and perceived stress by intervention**

|  | **Intervention (n = 18)** | | |
| --- | --- | --- | --- |
|  | **Control** | **Active** | **Mean difference active vs. control (95% CI)** |
| **Weight, kg** |  |  |  |
| Pre | 74.3 (66.7-83.2) | 72.3 (66.4-84.0) |  |
| Post | 72.4 (65.6-82.0)^1^ | 71.0 (65.5-82.6)^1^ | -0.0 (-0.4, 0.4)  P = 0.816 |
| **BMI, kg/m^2^** |  |  |  |
| Pre | 28.0 ± 3.7 | 28.0 ± 3.9 |  |
| Post | 27.6 ± 3.7^1^ | 27.5 ± 3.8^1^ | -0.0 (-0.2, 0.2)  P = 1.000 |
| **Body fat mass, kg** |  |  |  |
| Pre | 29.0 (23.8-36.8) | 29.5 (24.7-36.3) |  |
| Post | 27.9 (23.8-35.0)^1^ | 28.8 (24.2-35.9)^1^ | 0.3 (-0.1, 0.6)  P = 0.101 |
| **Waist circumference, cm** |  |  |  |
| Pre | 95.9 ± 10.6 | 96.9 ± 9.6 |  |
| Post | 95.1 ± 10.8 | 96.1 ± 9.4 | 0.1 (-1.9, 2.1)  P = 0.913 |
| **Hip circumference, cm** |  |  |  |
| Pre | 105.9 ± 7.9 | 105.8 ± 8.3 |  |
| Post | 106.3 ± 9.1 | 105.9 ± 9.1 | -0.2 (-1.2, 0.7)  P = 0.646 |
| **Waist-to-hip ratio** |  |  |  |
| Pre | 0.9 ± 0.1 | 0.9 ± 0.1 |  |
| Post | 0.9 ± 0.1 | 0.9 ± 0.1 | 0.0 (-0.0, 0.0)  P = 0.775 |
| **Energy expenditure from physical activity, kJ/day** |  |  |  |
| Pre | 2980 ± 1592 | 2660 ± 1394 |  |
| Post | 2823 ± 1272 | 2285 ± 1493 | -218 (-1035, 598)  P = 0.600 |
| **Perceived stress, raw score^2^** |  |  |  |
| Pre | 7.7 ± 5.4 | 7.1 ± 4.3 |  |
| Post | 8.0 ± 3.9 | 6.8 ± 4.0 | -0.5 (-3.0, 2.0)  P = 0.701 |
| Values are mean ± standard deviation or median (interquartile range) as indicated.  P-values for pre- vs. post-intervention comparison within the intervention group were obtained using Wilcoxon signed ranks test for non-normally distributed data and paired t test for normally distributed data.  ^1^P < 0.001 for pre- vs. post-intervention comparison within the intervention group.  ^2^Perceived stress raw scores were calculated using the Perceived Stress Scale. Scores can range from 0-40 (0-13 = low stress, 14-26 = moderate stress, 27-40 = high stress) (14).  Abbreviations: BMI, body mass index; CI, confidence interval; Pre, pre-intervention; Post, post-intervention | | | |
